# Mesenteric Venous Malformation With Subocclusive Symptoms: A Case Report

**DOI:** 10.1155/crgm/8396838

**Published:** 2025-11-29

**Authors:** Joseph Amara, Elio Mikhael, Santa El Helou, Jad Hachem, Rita Slim, César Yaghi

**Affiliations:** ^1^Department of Gastroenterology, Hôtel-Dieu de France University Hospital, Beirut, Lebanon; ^2^Faculty of Medicine, Université Saint-Joseph, Beirut, Lebanon; ^3^Department of General Surgery, Hôpital Saint-Joseph des Soeurs de la Croix, Mount-Lebanon, Dora, Lebanon

**Keywords:** hemangioma, intestinal obstruction, mesenchymal tumors, mesenteric tumors

## Abstract

Venous malformations, previously referred to as cavernous hemangiomas, can affect the gastrointestinal tract and rarely the mesentery. When symptomatic, it can cause pain, hematochezia, or less frequently obstructive symptoms. In this paper, we report the case of a 60-year-old man who presented to the emergency department for abdominal pain and subocclusive symptoms. A CT scan with intravenous contrast revealed a well-defined mesenteric mass measuring 3.5 × 3.2 cm in the pelvic region with a localized anterior panniculitis. A surgical resection was performed which led to resolution of obstructive symptoms, with the histopathological analysis confirming the diagnosis of mesenteric venous malformation.

## 1. Introduction

Venous malformations, previously referred to as cavernous hemangiomas, are benign vascular tumors that can be found everywhere in a human body but rarely in the gastrointestinal tract [1–3]. In the latter, it mainly affects the small intestine and usually presents in the form of hematochezia when symptomatic [4, 5]. In this paper, we report a rare case of mesenteric venous malformation with subocclusive symptoms.

## 2. Case

A 60-year-old Caucasian male presented to the emergency department with worsening abdominal pain and acute exacerbation of known constipation. His past medical history was significant for hypertension and dyslipidemia, and he is an active smoker. He had no previous surgical history. He had recently been complaining of recurrent postprandial hypogastric pain partially resolving after defecation and partially calmed by antispasmodic. He denied the presence of melena or hematochezia. His last bowel movement was 2 days prior to admission. There was no weight loss reported.

On examination, the patient's vital signs were normal. His abdomen was soft but mildly distended. The abdominal sounds were hyperactive. Deep palpation, especially in the hypogastric and pelvic regions, elicited tenderness. The hernial orifices were intact, and the scrotum appeared normal. The digital rectal examination was unpainful and revealed Type 1 hemorrhoids. The rest of the systemic examination was normal.

**Figure 1:**
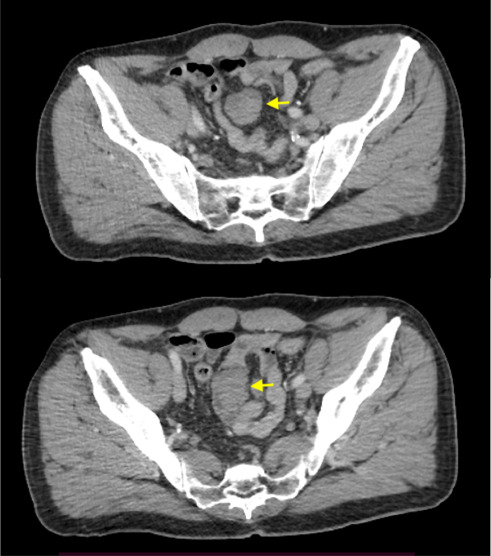
CT scan with intravenous contrast revealed a well-defined mesenteric mass measuring 3.5 × 3.2 cm in the pelvic region (yellow arrow).

Laboratory evaluation including complete blood count, liver, pancreatic, and metabolic panels was unremarkable excepting mildly elevated CRP 10 mg/L (normal range < 3 mg/L) and ferritin levels at 287 ng/mL (normal range 21.8–274.5 ng/mL). An abdominal computed tomography (CT scan) with intravenous contrast revealed a well-defined mesenteric mass measuring 3.5 × 3.2 cm in the pelvic region with a localized anterior panniculitis (Figure 1). MRI was not performed due to unavailability in an acute setting and due to patient's claustrophobia.

**Figure 2:**
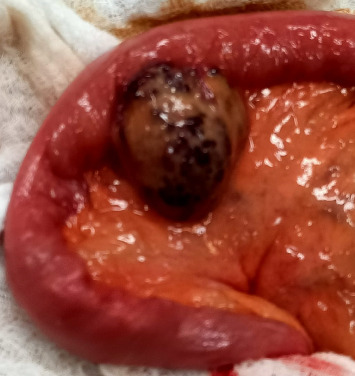
Surgical piece of an intestinal resection that is 9 cm long, centered around a 4-cm subserosal nodule containing hemorrhagic content and thrombi.

**Figure 3:**
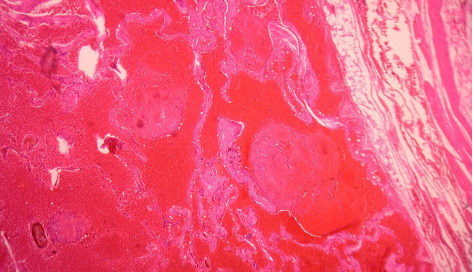
Venous malformation. Well-circumscribed intramuscular nodule containing vascular cavities with endothelial lining (caverns) filled with a hemorrhagic content and microthrombi. There is no cellular atypia.

In the context of a mesenteric mass with subocclusive symptoms, surgery was warranted. An exploratory laparoscopy showed a nodular mesenteric purple mass, 4 m proximal to the ileocecal valve (Figure 2). The mesenteric mass along with the involved part of the jejunum (20 cm) were resected, and a side-to-side anastomosis was performed. The postoperative follow-up was uneventful. On pathology, the resected lesion revealed a well-circumscribed venous malformation consistent with prior definitions of cavernous hemangioma, composed of dilated vascular channels lined by endothelium and filled with blood and microthrombi (Figure 3).

The patient was discharged home on the third day postoperative. Follow-up evaluations at 1 week and 1 month after surgery were unremarkable, with resolution of the previous symptoms. He was last seen 6 months postoperatively and remained asymptomatic.

## 3. Discussion

We herein report a case of a mesenteric venous malformation presenting with subocclusive symptoms. Venous malformations are benign and can develop anywhere in the body although it is rarely found in the gastrointestinal tract [1, 3]. In fact, it represents less than 1% of all gastrointestinal tumors [4]. It is found most frequently in the small bowel, and particularly in the jejunum, however, mesenteric involvement is rare [6]. In the literature, the mean age at diagnosis is 41 years old with a female-to-male ratio at 1.55 [7].

When symptomatic, the most common manifestation is gastrointestinal bleeding or anemia, but abdominal pain, dyspepsia, intussusception, and intestinal perforation can be seen [5]. There have been previous reports of venous malformations presenting as small bowel obstruction as demonstrated in this case [6, 7]. In our case, the patient presented with subocclusive symptoms.

Many imaging techniques have been used to diagnose venous malformations. The CT scan has a limited role in the diagnosis but can be very useful to determine the location and size and to plan a subsequent therapeutic act [5]. In the nonenhanced CT scan, venous malformations appear as polypoid or nodular lesions into the lumen of the intestine, with or without vascular calcifications (phleboliths) [1]. With intravenous contrast, they are characterized by peripheral nonhomogeneous enhancement of lesion with thickening of the intestine mucosa [4]. In our case, the CT scan set the diagnosis of an intestinal obstruction secondary to a lesion and aided in surgical planning.

Other imaging techniques can be used as well, such as the MRI and scintigraphy. On MRI, venous malformations appear as a high signal intensity lesion on T2-weighted sequences and low intensity on diffusion-weighted and T1-weighted sequences [8]. In our case, an MRI was not performed due to its limited availability in emergency settings and the patient's claustrophobia. In addition, given that MRI does not certify the diagnosis and given the subocclusive presentation [8], surgical intervention was deemed necessary. Endoscopy (EGD, colonoscopy, enteroscopy, or capsule endoscopy) seems to be an efficient diagnostic method of gastrointestinal venous malformations (EGD, colonoscopy or enteroscopy) [5]. However, given that this patients' disease is mesenteric and not endoluminal, endoscopic evaluation was not necessary.

The diagnosis of venous malformations relies mainly on pathology. They usually originate from the submucosal layer and might extend outward through the musculosa and the serosa [6]. The initial pathology report identified the lesion as a cavernous hemangioma. There are three types of hemangiomas: cavernous being the most common (characterized by the presence of large, irregular blood-filled sinuses or spaces), capillary (characterized by a proliferation of thin-walled vessels not always filled with blood), and mixed [9–11]. However, since the introduction of updated nomenclature in 2018, this condition is now classified under the broader term vascular malformation [2].

The differential diagnosis of a mesenteric mass is broad. Mesenteric masses can be categorized into cystic masses (including cystic lymphangioma, mesothelial cyst, mesothelioma, mucinous cyst, pseudocyst, and desmoid cyst) and solid masses (including lymphoma, stromal tumors, lipoma, liposarcoma, desmoid tumors, mesenteric panniculitis, inflammatory pseudotumors, and fibroma) [12]. Venous malformations are a rare cause of mesenteric tumors, with approximately 20 cases reported in the literature. Clinical, laboratory, and imaging data are rarely definitive for diagnosis [13]. Histologic confirmation via surgical resection or fine-needle biopsy is almost always required [12].

The mainstay of treatment for venous malformations is surgical resection. They do not spread to lymph nodes or distant organs; therefore, local resection is sufficient [14]. However, when there is a high suspicion of malignancy (ascites, peritoneal deposits, presence of lymphadenopathies), a frozen section procedure could be done before resection [7]. For a lesion that presents with obstructive symptoms, an exploratory laparoscopy is warranted as is the case in our patient [7]. After surgical excision, our patient had an unremarkable follow-up with notable clinical improvement. Endoscopic therapy has been scarcely used in the literature and seems to be effective for small endoluminal lesions only [14]. Therefore, it is not applicable in this case.

## 4. Conclusion

Venous malformations are benign vascular tumors that rarely involve the mesentery. Intestinal obstruction is a rare but possible presentation and typically requires surgical resection. As imaging findings are often nonspecific, definitive diagnosis relies on histopathological examination.
